# Socio-economic inequalities in body mass index among preschool children: do sports programs in early childhood education and care centers make a difference?

**DOI:** 10.3389/fpubh.2023.1079871

**Published:** 2023-06-22

**Authors:** Alena Mayer, Raphael M. Herr, Thomas Klein, Eva Wiedemann, Katharina Diehl, Stephanie Hoffmann, Miriam Blume, Dennis Jepsen, Leonie Sundmacher, Marike Andreas, Sven Schneider

**Affiliations:** ^1^Center for Preventive Medicine and Digital Health (CPD), Medical Faculty Mannheim, Heidelberg University, Mannheim, Germany; ^2^Department of Medical Informatics, Biometry and Epidemiology, Friedrich-Alexander-Universität Erlangen-Nürnberg (FAU), Erlangen, Germany; ^3^Max-Weber-Institute of Sociology, Faculty of Economics and Social Sciences, Heidelberg University, Heidelberg, Germany; ^4^Department of Public Health, Brandenburg University of Technology Cottbus-Senftenberg, Cottbus, Senftenberg, Germany; ^5^Department of Epidemiology and Health Monitoring, Robert Koch Institute, Berlin, Germany; ^6^Institute of Medical Sociology, Medical Faculty, Martin-Luther University Halle-Wittenberg, Halle (Saale), Germany; ^7^Chair of Health Economics, Technical University of Munich, München, Germany

**Keywords:** BMI, children, meso-level, sport, early childhood education and care center, pre-school, health equalities

## Abstract

**Background:**

Overweight in childhood is considered to be one of the most serious public health challenges. Many studies have investigated individual-level determinants of children's body mass index (BMI), yet studies exploring determinants at the meso-level are sparse. The aim of our study was to examine how a sports focus at early childhood education and care (ECEC) centers moderates the effect of parental socio-economic position (SEP) on children's BMI.

**Methods:**

We used data from the German National Educational Panel Study and included 1,891 children (955 boys and 936 girls) from 224 ECEC centers in our analysis. Linear multilevel regressions were used to estimate the main effects of family SEP and the ECEC center sports focus, as well as their interaction, on children's BMI. All analyses were stratified by sex and adjusted for age, migration background, number of siblings, and employment status of parents.

**Results:**

Our analysis confirmed the wellknown health inequalities in childhood overweight with a social gradient toward a higher BMI for children from lower SEP families. An interactive effect between family SEP and ECEC center sports focus was found. Boys with low family SEP not attending a sports-focused ECEC center had the highest BMI among all boys. In contrast, boys with low family SEP attending a sports-focused ECEC center had the lowest BMI. For girls, no association regarding ECEC center focus or interactive effects emerged. Girls with a high SEP had the lowest BMI, independent of the ECEC center focus.

**Conclusion:**

We provided evidence for the gender-specific relevance of sports-focused ECEC centers for the prevention of overweight. Especially boys from low SEP families benefited from a sports focus, whereas for girls the family's SEP was more relevant. As a consequence, gender differences in determinants for BMI at different levels and their interaction should be considered in further research and preventive measures. Our research indicates that ECEC centers may decrease health inequalities by providing opportunities for physical activity.

## 1. Introduction

Worldwide, the proportion of children being overweight has notably increased within the last decades (1). Overweight is defined as abnormal or excessive fat accumulation, which is associated with a higher chance of subsequent overweight, disability, and premature death in adulthood (2, 3). In addition, overweight children suffer from respiratory problems, hypertension, early signs of cardiovascular diseases, and psychological health problems (2, 4–6).

Overweight in childhood is considered one of the twentyfirst century's most serious public health challenges, which is arising from complex interactions between biological, behavioral, socio-environmental, and basic environmental factors (5–9). Despite major efforts to promote weight reduction, early childhood overweight has reached epidemic proportions in high-income countries (10). In 2019, ~38 million children under the age of 5 years were classified as overweight or obese worldwide (2). In Germany, a representative study has revealed that the percentage of overweight children is 10.8% for 3–6-year-old girls and 7.3% among boys. The prevalence of obesity among 3–6-year-old girls and boys is 3.2% and 1.0%, respectively (11).

In addition to the factors mentioned above, health in early childhood also depends on the socio-economic position (SEP) of the family, which is usually defined by parental education, occupation, and household income (12). In high-income countries, epidemiological studies have consistently shown that children with socio-economic disadvantages (i.e., low family SEP) have disproportionately poorer health outcomes than socio-economic advanced children and are more likely to be affected by childhood overweight (11, 13–16). Since health-related attitudes and behaviors formed at an early age are often carried into adulthood, health inequalities during childhood and adolescence might provide the foundation for health inequalities across the life course (17–20). Therefore, childhood and adolescence are particularly suitable time frames for health prevention and promotion (13, 21, 22).

One of the most effective interventions for childhood overweight is physical activity (23, 24). Preschool age is considered a critical window for the development of young children's physical activity habits (25). Scientists agree that children in these early years should be abundantly physically active through structured and unstructured play (26–28). Movement, play, and sports are of great importance in early childhood education and care (ECEC) centers as they function as a central socialization instance and have a formative influence on the health behavior of preschool children (29–32). Moreover, a sports focus of ECEC centers in the form of specific physical activity programs enjoys great popularity with parents and educators in Germany (33). Thus, ECEC centers represent feasible settings for health interventions, as 92% of children under 6 years of age are cared for in a daycare center in Germany (34). This study, therefore, aimed to examine the independent and interaction effects of family SEP and an ECEC center sports focus on the body mass index (BMI) of preschoolers. As significant gender differences could be expected in the relevance of these factors, all analyses were stratified by sex (35, 36).

## 2. Material and methods

Secondary data analysis was performed using data from the German National Educational Panel Study (NEPS) (37) of the Leibniz Institute for Educational Trajectories (LIfBi) at the University of Bamberg. The NEPS is a nationwide representative study with a multi-cohort sequence design. The main objective of the NEPS is the collection of life span data on the development of competencies, educational processes, educational decisions, and returns to education in different contexts. Surveys were carried out with children and their parents, as well as with educators and the institution heads of the ECEC centers. The clustering within ECEC centers makes multilevel analyses to consider the meso-level possible (38). In this study, we used the first wave of Starting Cohort 2 “Kindergarten” (SC2). Of the 2,996 children, 1,891 children (955 boys and 936 girls) from 224 ECEC centers had valid data on relevant variables (see below) and were included in our analyses.

### 2.1. Outcome: children's BMI

The parents gave information on the weight and height of their children. The BMI was defined by the standard formula: body weight in kilograms divided by the square of its height in meters (kg/m^2^). Implausible values were excluded (BMI < 10 or > 100). For children, age needs to be considered since the relationship between body size and weight changes due to growth. Thus, we adjusted all analyses for age (2, 39). As boys and girls also have different BMIs, analyses were stratified for gender.

### 2.2. Independent variables

#### 2.2.1. Family SEP

Family SEP was included by tertiles (low, middle, and high SEP) from the highest occupational status of the mother or the father in the family, measured by the International Socio-Economic Index of Occupational Status (ISEI 08). The ISEI is an established international index that measures socio-economic status based on educational attainment, occupation, and income (40, 41).

#### 2.2.2. Meso-level: ECEC center with a sports focus

The information on whether an ECEC center had a sports focus or not was given by the institution heads by their answer to the following question with either yes or no: “Does your facility focus on a special field of activity (motor skill activity/movement) in addition to normal pedagogic work?” According to study information, also given in the interviews, an ECEC center was designated as a sports-focused ECEC center, “…, if an essential portion of everyday kindergarten life is used to promote this focus on a regular basis and the staff used for that purpose has the appropriate qualification” (37).

### 2.3. Covariates

The gender and the age of the child were given by the respondents (37). The respondents also stated the number of siblings in the household (categorized into none, one, two, or more) and their own employment status (full-time, part-time, side job, and unemployed). Migration background was coded if German was not the predominant language spoken at home.

### 2.4. Analyses

All analyses were stratified by sex, and all tests were considered significant at a *p*-value of < 0.05. All analyses were performed in February 2022 using Stata SE (version 14). The sample characteristics of girls and boys and according to SEP tertiles were compared using the chi-square test or the F-test. Linear multilevel regression analyses (level 1 = children, level 2 = ECEC center) were conducted to calculate the main effects of family SEP and ECEC center sports focus, as well as their interaction, on children's interval-scaled BMI. In addition, predictive margins (delta method) were estimated. To standardize results, BMI was Z-transformed, and all analyses were adjusted for age, migration background, number of siblings (none, one, two, or more), and employment status of the interviewed (full-time, part-time, side job, and unemployed).

## 3. Results

Of the total 1,891 children from the 224 ECEC centers, 936 were girls and 955 were boys, representing a sufficient sample size for multilevel investigations (42, 43). Of these, 983 children were enrolled in an ECEC center with a sports focus. Children were 5 years old on average (mean = 4.98, SD = 0.34). Table 1 shows an overview of all demographic variables stratified by sex. Boys had a significantly higher mean BMI than girls (boys: mean = 15.59, SD = 3.52; girls: mean = 15.17, SD = 2.44; *p* = 0.0025). There were no significant differences in the other independent variables.

**Table 1:** Study population description stratified by sex.

	**Total (*****n*** = **1,891)**		**Boys (*****n*** = **955)**		**Girls (*****n*** = **936)**			
	**mean/%**	**SD/** ***n***	**mean/%**	**SD/** ***n***	**mean/%**	**SD/** ***n***	**Test value**	***P*** **-value**
**Micro-level**								
BMI	15.39	3.04	15.59	3.52	15.17	2.44	9.17	0.0025
**Family SEP**								
Low	32.63	617	31.41	300	33.87	317	1.599	0.450
Middle	35.91	679	37.07	354	34.72	325		
High	31.46	595	31.52	301	31.41	294		
**Covariates**								
Age	4.98	0.34	4.98	0.33	4.98	0.35	0.538	0.463
Migration background (yes)	10.74	203	9.74	93	11.75	110	2.001	0.157
**Employment status respondent**								
Full-time	19.46	368	19.58	187	19.34	181	1.400	0.705
Part-time	41.57	786	41.57	397	41.56	389		
Side job	8.14	154	8.80	84	7.48	70		
Unemployed	30.83	583	30.05	287	31.62	296		
**Siblings**								
No sibling	22.37	423	21.68	207	23.08	216	0.759	0.684
1 sibling	51.51	974	51.52	492	51.5	482		
2+ siblings	26.12	494	26.81	256	25.43	238		
**Meso-level**								
ECEC center: sports focus (yes)	51.98	983	50.99	487	52.99	496	0.755	0.385

Test value, F-test for continuous variables and the chi-square test for categorical variables.

Table 2 depicts children's demographic variables stratified for each SEP tertile. BMI was the highest in the low-family SEP tertile and the lowest in the high-family SEP tertile. Slightly more than half of the examined children in the sample attended an ECEC center with a sports focus (51.98%). There were no differences in the attendance rate regarding the SEP (p = 0.643).

**Table 2:** Main outcome and correlates at the micro- and meso-level according to socio-economic position (family SEP).

	**Family SEP high**		**Family SEP middle**		**Family SEP low**			
	**mean/%**	**SD/** ***n***	**mean/%**	**SD/** ***n***	**mean/%**	**SD/** ***n***	**Test value**	***P*** **-value**
**Micro-level**								
BMI	15.11	2.03	15.16	1.95	15.90	4.45	13.36	< 0.001
**Covariates**								
Age	4.94	0.33	4.99	0.34	5.02	0.35	2.97	0.227
Migration background (yes)	5.88	35	7.51	51	18.96	117	65.57	< 0.001
**Employment status respondent**								
Full-time	18.15	108	20.62	140	19.45	120	50.22	< 0.001
Part-time	49.75	296	42.86	291	32.25	199		
Side job	5.21	31	9.13	62	9.89	61		
Unemployed	26.89	160	27.39	186	38.41	237		
**Siblings**								
No sibling	18.82	112	22.24	151	25.93	160	25.43	< 0.001
1 sibling	55.8	332	54.93	373	43.6	269		
2+ siblings	25.38	151	22.83	155	30.47	188		
**Meso-level**								
ECEC center: sports focus (yes)	53.28	317	50.66	344	52.19	322	0.884	0.643

Test value, F-test for continuous variables and Chi^2^ test for the categorical variable.

**Figure 1:**
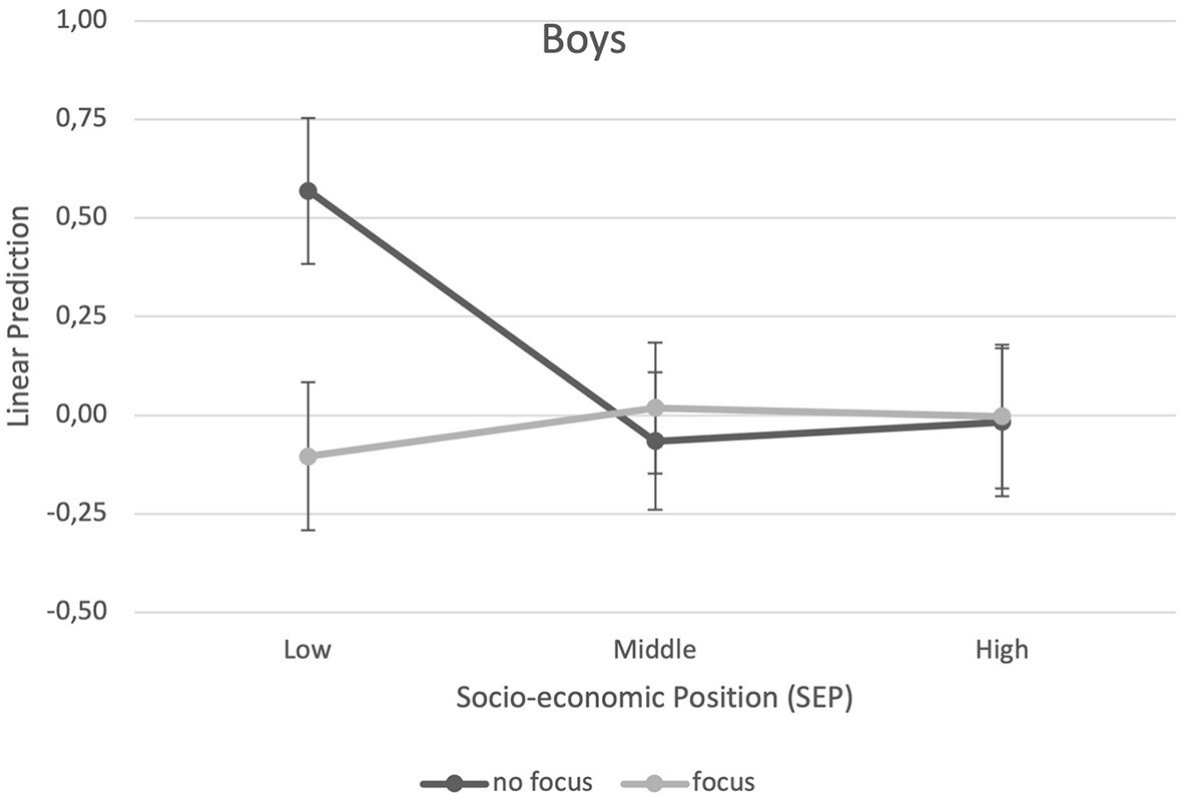
Predictive margins of ECEC center sports focus (yes vs. no) and socio-economic position (family SEP) on BMI for boys.

**Figure 2:**
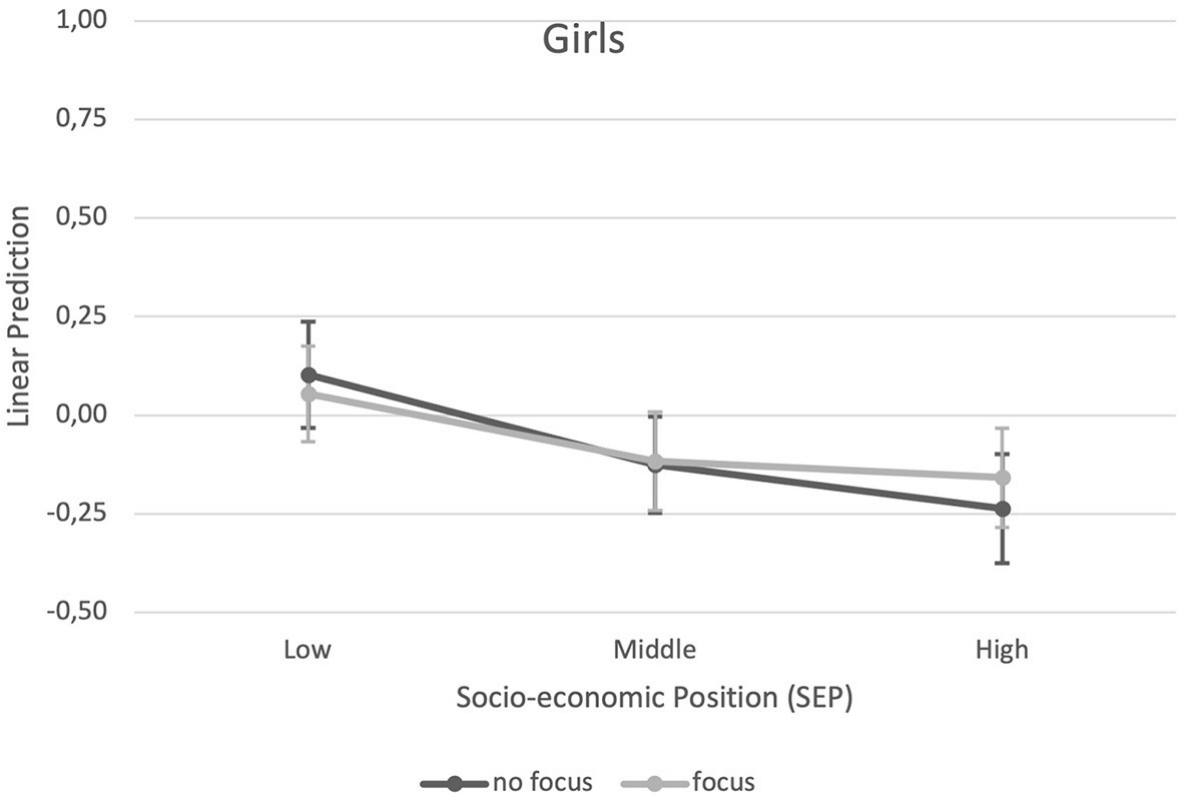
Predictive margins of ECEC center sports focus (yes vs. no) and socio-economic position (family SEP) on BMI for girls.

The results of the multilevel analysis of the main and interaction effects of family SEP and ECEC center focus on BMI are presented in Table 3. For boys, a significant main effect indicated a generally lower BMI in the middle SEP tertile and in the highest SEP tertile compared to the lowest SEP category. Another main effect showed that boys attending an ECEC center with a sports focus had a lower BMI than boys who do not attend a center with a sports focus. In addition, interactive effects between family SEP and ECEC center sports focus occurred (Table 3). Considering the predictive margins (Figures 1, 2), boys with low family SEP not attending a sports focus ECEC center had the highest BMI, while boys with low family SEP attending a sports focus ECEC center had the lowest BMI. For girls, a significant main effect revealed a generally lower BMI in the middle and high family SEP tertiles compared to the lowest SEP tertile. No association of ECEC center focus or interactive effect emerged for girls. Girls with high family SEP had the lowest BMI in both ECEC center types (with or without sports focus).

**Table 3:** Main and interaction effects of ECEC center sports focus and socio-economic position (family SEP) on BMI for boys and girls.

	**Boys (*****n*** = **955, centers** = **223)**			**Girls (*****n*** = **936, centers** = **224)**		
	**Coef**.	**Std. Err**.	***P*** **-value**	**Coef**.	**Std. Err**.	***P*** **-value**
**Main effects**						
**Family SEP (ref: Low)**						
Middle	−0.635	0.128	< 0.001	−0.228	0.093	0.014
High	−0.586	0.134	< 0.001	−0.339	0.099	< 0.001
**Employment status respondent (ref: full-time)**						
Part-time	0.057	0.102	0.574	0.075	0.073	0.306
Side job	−0.050	0.151	0.740	−0.049	0.114	0.666
Unemployed	−0.092	0.109	0.401	−0.039	0.079	0.620
Age (years)	−0.065	0.039	0.096	0.006	0.027	0.833
Migration background (yes)	0.507	0.127	< 0.001	0.093	0.085	0.273
**Siblings (ref: no)**						
1 sibling	0.151	0.096	0.116	0.007	0.067	0.915
2+ siblings	0.291	0.110	0.008	0.126	0.079	0.111
ECEC center: sports focus (yes)	−0.067	0.133	< 0.001	−0.048	0.092	0.601
**Interaction effect**						
Middle SEP × ECEC sports focus	0.758	0.179	< 0.001	0.057	0.129	0.655
High SEP × ECEC sports focus	0.687	0.186	< 0.001	0.127	0.132	0.337

## 4. Discussion

The analysis of 1,891 German preschool children revealed that the BMI of the preschoolers was related to the family SEP. Our results show that boys and girls from lower SEP families had a higher BMI on average. For boys, the sports focus of the ECEC center also played a role. We found that the association between SEP and BMI among boys was moderated by the sports focus of ECEC centers. Visiting an ECEC center with a sports focus appears especially health-promoting for boys from a weaker socio-economic background.

Our analysis showed a social gradient toward higher BMI for socio-economically disadvantaged children for both sexes. However, we found evidence for the relevance of ECEC center sports focus for BMI for boys only. Therefore, it might be speculated that for boys' factors outside the family might be relevant concerning BMI, whereas for girls, the family SEP seems to play a more important role (44). Our finding is all the more surprising considering that girls are known to have more sedentary lifestyles and lower levels of physical activity than boys (45). For this reason, it would be expected that girls within an ECEC will particularly benefit from a specific and consolidated curriculum for the promotion of physical activity.

Regarding socio-economic inequalities in health, surprisingly little is known about factors located at the meso-level since previous research on preschool children's BMI has focused predominantly on the micro level (46). One explanation might be the complexity and dynamics of the system studied (47, 48). As a consequence, there is little empirical knowledge about the role of institutions that form a central link between the individual and the macro level in the emergence of patterns of health inequalities in the developmental stages from early childhood to early adulthood (49, 50). This hinders the effective design of institutional interventions to promote better health, which is especially important, as childhood overweight is related to a higher risk of illness in adulthood (e.g., cardiovascular diseases or chronic illness), stigma, reduced self-esteem (51, 52), and a higher psychological stress perception (53). Certainly, the negative consequences of overweight on health do not occur as late as adulthood. Gender differences in the relationship between overweight and social inequality increase with age (54) and can be explained by sex differences (e.g., hormonal balance and neurological factors) and gender differences (e.g., gender-based stereotypes and related parental expectations that influence parenting) (55). Further studies should focus on how these biological and socio-cultural factors interact with the BMI and SEP of children already in preschool age and how ECEC centers can impact these effects in a positive way to prevent health and gender inequalities during the life course.

In addition to the sports focus of the ECEC center, other relevant factors might also be conceivable. For example, the composition of the group, the experience and expertise of the teachers, and the equipment available at the ECEC center might play relevant roles. Thus, further research might reveal other relevant ECEC characteristics.

In addition, further research might investigate which type of supply (e.g., sports courses, swimming, physical activity offers, enrichment of outdoor areas, equipment of the indoor area, or the sheer size of the facility) is related to the greatest increase in physical activity time. In this context, it would be also interesting to investigate whether physical aspects (e.g., area, number of playground equipment, and attractiveness of playground equipment) and social aspects (e.g., staff, qualification, and attitude of staff) interact.

In ECEC centers, a suitable physical activity environment should be created so that preschoolers can develop physical activities. In addition, evidence exists that the amount of physical activity in preschool age positively influences the amount of time people are active in adulthood (56). Wellqualified pedagogical staff promoting physical activity should therefore instruct the children's exercises (57).

It appears advisable that overweight prevention and treatment interventions should address the most disadvantaged groups to not further exacerbate inequalities in weight (58). Effectively tackling overweight, therefore, requires a “proportionate universalism” (59), whereby interventions are delivered at the level that meets the need across the social gradient. In our study, boys in the lowest family SEP tertile seem to benefit most from an ECEC center with a focus on sports. Accordingly, ECEC centers could play a critical role in addressing health inequalities regarding BMI, at least for boys.

All analyses were repeated with an alternative calculation for the BMI in children (60). The standard deviation score of the BMI (SDS BMI) was calculated based on representative data for Germany (61). The SDS BMI is standardized for age and gender and transformed to the value range of a standard normal distribution. This sensitive analysis generally yielded comparable results.

Several limitations have to be considered. Since this was a secondary data analysis, the possibilities for capturing individual and meso-level aspects were limited. In particular, further characteristics of the ECEC would be relevant here. These are usually differentiated into physical (sports equipment, movement areas, and architecture), economic (kindergarten fees), political (curriculum, rules, and timetables), and socio-cultural (attitudes and social norms) characteristics. Whether a child moves a lot or little in an ECEC center is then determined by a complex interplay of these characteristics (62). Another limitation refers to the outcome. The BMI represents a simple index of weight-for-height that is commonly used to classify overweight and obesity. Other measures might appear more suitable in the age of preschoolers, for example, skinfold thickness measurement or waist-to-hip ratio (2). Nevertheless, the BMI has the advantage that it is easy to measure and can therefore also be used with preschool children. However, this indicator does not consider the typical growth spurts in the preschool age group, which can lead to a statistical overshadowing of possible effects of physical activity promotion. In addition, improper body posture and body deformities, which are usually associated with increased BMI and obesity, should be considered in further studies. Furthermore, a selection effect cannot be ruled out; children who are already more active might be more likely to be enrolled in sports-focused ECEC centers. This means that the variable BMI is potentially endogenous which could bias the analysis. Another source of bias might be in the measurement of height and weight to calculate BMI. As in other large-scale population-based studies, this study used parent-rated data to assess height and weight. These estimates appear, however, less sensitive for underweight and overweight and might bias results (63, 64). Further studies are therefore needed to confirm the findings by applying professionally measured data for weight and height. In addition, it was not considered, whether the children examined lived with only one parent as there is evidence that children of single parents are more physically active and play outside a lot more (65). Future research could also take this aspect into consideration.

In conclusion, this study revealed the importance of daily physical activity for boys regarding their BMI in ECEC centers, especially for boys with low family SEP. Particularly boys from socio-economically disadvantaged families seem to benefit from visiting an ECEC center with a sports focus. However, for girls, no association of the ECEC center sports focus or interactive effects with SEP with BMI was found. Taken together, our analysis indicates that attempts to reduce the social gradient in BMI should take the gender as well as the characteristics of the ECEC center into account as they play an independent and interactive role.

## Data availability statement

Publicly available datasets were analyzed in this study. This data can be found here: https://www.neps-data.de/Datenzentrum/Datenzugangswege.

## Ethics statement

The studies involving human participants were reviewed and approved by a special data protection and security officer of the NEPS. Written informed consent to participate in this study was provided by the participants' legal guardian/next of kin.

## Author contributions

RH analyzed the data. RH and AM interpreted the data. AM, RH, EW, and SS drafted the manuscript. SH, MB, KD, DJ, LS, TK, and MA critically revised the manuscript. All authors read and approved the final manuscript.

## References

[1] Abarca-Gómez L Abdeen ZA Hamid ZA Abu-Rmeileh NM Acosta-Cazares B Acuin C. Worldwide trends in body-mass index, underweight, overweight, and obesity from 1975 to 2016: a pooled analysis of 2416 population-based measurement studies in 128·9 million children, adolescents, and adults. The Lancet. (2017) 390:2627–42. 10.1016/S0140-6736(17)32129-3 29029897 PMC5735219

[2] World Health Organization. Obesity and Overweight, Fact Sheet. (2021). Available online at: https://www.who.int/news-room/fact-sheets/detail/obesity-and-overweight (accessed May, 2022).

[3] Helba M Binkovitz LA. Pediatric body composition analysis with dual-energy X-ray absorptiometry. Pediatr Radiol. (2009) 39:647–56. 10.1007/s00247-009-1247-0 19415261

[4] Gahagan S. Child and adolescent obesity. Curr Probl Pediatric Adolesc Health Care. (2004) 34:6–43. 10.1016/j.cppeds.2003.09.001 14685151

[5] Ang YN Wee BS Poh BK Ismail MN. Multifactorial influences of childhood obesity. Curr Obesity Rep. (2012) 2:10–22. 10.1007/s13679-012-0042-7

[6] Ebbeling CB Pawlak DB Ludwig DS. Childhood obesity: public-health crisis, common sense cure. Lancet. (2002) 360:473–82. 10.1016/S0140-6736(02)09678-2 12241736

[7] Budd GM Hayman LL. Childhood obesity: determinants, prevention, and treatment. J Cardiovasc Nurs. (2006) 21:437–41. 10.1097/00005082-200611000-00005 17293732

[8] Fairclough SJ Boddy LM Hackett AF Stratton G. Associations between children's socioeconomic status, weight status, and sex, with screen-based sedentary behaviours and sport participation. Int J Pediatr Obes. (2009) 4:299–305. 10.3109/17477160902811215 19922045

[9] Jia P. Obesogenic environment and childhood obesity. Obesity Rev. (2021) 22:e13158. 10.1111/obr.13158 33258179

[10] Goldfield GS Harvey A Grattan K Adamo KB. Physical activity promotion in the preschool years: a critical period to intervene. Int J Environ Res Public Health. (2012) 9:1326–42. 10.3390/ijerph9041326 22690196 PMC3366614

[11] Schienkiewitz A Brettschneider A Damerow S Rosario AS. Ü*bergewicht und Adipositas im Kindes- und Jugendalter in Deutschland- Querschnittergebnisse aus KiGGS Welle 2 und Trends2018*.

[12] Hoffmann S Sander L Wachtler B Blume M Schneider S Herke M. Moderating or mediating effects of family characteristics on socioeconomic inequalities in child health in high-income countries—a scoping review. BMC Public Health. (2022) 22:338. 10.1186/s12889-022-12603-4 35177014 PMC8851861

[13] OECD. Obesity and the Economics of Prevention (2010).

[14] Howe LD Tilling K Galobardes B Smith GD Ness AR Lawlor DA. Socioeconomic disparities in trajectories of adiposity across childhood. Int J Pediatr Obes. (2011) 6:e144–53. 10.3109/17477166.2010.500387 20860432 PMC5102325

[15] Shrewsbury V Wardle J. Socioeconomic status and adiposity in childhood: a systematic review of cross-sectional studies 1990-2005. Obesity (Silver Spring). (2008) 16:275–84. 10.1038/oby.2007.35 18239633

[16] Pillas D Marmot M Naicker K Goldblatt P Morrison J Pikhart H. Social inequalities in early childhood health and development: a European-wide systematic review. Pediatr Res. (2014) 76:418–24. 10.1038/pr.2014.122 25122581

[17] Kuntz B Lampert T. Wie gesund leben jugendliche in Deutschland? Ergebnisse des Kinder- und Jugendgesundheitssurveys (KiGGS). Gesundheitswesen. (2013) 75:67–76. 10.1055/s-0032-1311620 22664796

[18] Lampert T. Frühe Weichenstellung: Zur Bedeutung der Kindheit und Jugend fur die Gesundheit im späteren Leben. Bundesgesundheitsblatt Gesundheitsforschung Gesundheitsschutz. (2010) 53:486–97. 10.1007/s00103-010-1055-6 20376416

[19] Blume M Rattay P Hoffmann S Spallek J Sander L Herr R. Health inequalities in children and adolescents: a scoping review of the mediating and moderating effects of family characteristics. Int J Environ Res Public Health. (2021) 18:15. 10.3390/ijerph18157739 34360031 PMC8345625

[20] Case A Fertig A Paxson C. The lasting impact of childhood health and circumstance. J Health Econ. (2005) 24:365–89. 10.1016/j.jhealeco.2004.09.008 15721050

[21] Jia P. Spatial lifecourse epidemiology. The Lancet Planetary Health. (2019) 3:e57–e9. 10.1016/S2542-5196(18)30245-6 30797406

[22] Pérez-Escamilla R Kac G. Childhood obesity prevention: a life-course framework. Int J Obes Suppl. (2013) 3:S3–s5. 10.1038/ijosup.2013.2 25018875 PMC4089584

[23] Avila C Holloway AC Hahn MK Morrison KM Restivo M Anglin R. An overview of links between obesity and mental health. Current Obesity Reports. (2015) 4:303–10. 10.1007/s13679-015-0164-9 26627487

[24] Gmeiner MS Warschburger P. Psychotherapie bei juveniler Adipositas: gerechtfertigt und sinnvoll? Psychotherapeut. (2021) 66:16–22. 10.1007/s00278-020-00474-2

[25] Muennig P Schweinhart L Montie J Neidell M. Effects of a prekindergarten educational intervention on adult health: 37-year follow-up results of a randomized controlled trial. American journal of public health. (2009) 99:1431–7. 10.2105/AJPH.2008.148353 19542034 PMC2707464

[26] Stevens J Murray DM Baggett CD Elder JP Lohman TG Lytle LA. Objectively assessed associations between physical activity and body composition in middle-school girls: the Trial of Activity for Adolescent Girls. Am J Epidemiol. (2007) 166 11:1298–305. 10.1093/aje/kwm202 17855391 PMC2150740

[27] Pietiläinen KH Kaprio JA Borg P Plasqui G Yki-Järvinen H Kujala UM. Physical inactivity and obesity: a vicious circle. Obesity. (2008) 16. 10.1038/oby.2007.72 18239652 PMC2249563

[28] Ward DS Vaughn A McWilliams C Hales D. Interventions for increasing physical activity at child care. Med Sci Sports Exerc. (2010) 42:526–34. 10.1249/MSS.0b013e3181cea406 20068495

[29] McGrady ME Mitchell MJ Theodore SN Sersion B Holtzapple E. Preschool participation and bmi at kindergarten entry: the case for early behavioral intervention. J Obes. (2010) 2010:407. 10.1155/2010/360407 20721345 PMC2915775

[30] Wartha O Steinacker JM Kobel S. Gesundheitsförderung an baden-württembergischen Kindertageseinrichtungen. Prävention und Gesundheitsförderung. (2018) 14:53–9. 10.1007/s11553-018-0647-0

[31] Bower JK Hales DP Tate DF Rubin DA Benjamin SE Ward DS. The childcare environment and children's physical activity. Am J Prev Med. (2008) 34:23–9. 10.1016/j.amepre.2007.09.022 18083447

[32] Hawkins SS Law C. A review of risk factors for overweight in preschool children: A policy perspective. Int J Pediatric Obesity. (2006) 1:195–209. 10.1080/17477160600943351 17907326

[33] De Bock F Fischer JE. Gesundheitsförderung im Kindergarten: Evaluation des Programms “Komm mit in das gesunde Boot” der Baden-Württemberg Stiftung in Kindergärten in Baden-Württemberg. (2011).

[34] Destatis. Kindertagesbetreuung: Betreuungsquote von Kindern unter 6 Jahren nach Bundesländern. (2021). Available online at: https://www.destatis.de/DE/Themen/Gesellschaft-Umwelt/Soziales/Kindertagesbetreuung/Tabellen/betreuungsquote.html/jsessionid=F9CDD773EDA7C20B5EE6D447D56E7196.live741 (accessed June, 2022).

[35] Yamamoto S Becker S Fischer J De Bock F. Sex differences in the variables associated with objectively measured moderate-to-vigorous physical activity in preschoolers. Prev Med. (2011) 52:126–9. 10.1016/j.ypmed.2010.11.014 21130113

[36] Gilligan C. In A Different Voice: Psychological Theory and Women's Development. (1982). p. 220.6349031

[37] NEPS-Netzwerk. Nationales Bildungspanel, Scientific Use File der Startkohorte Kindergarten. Leibniz-Institut für Bildungsverläufe (LIfBi), Bamberg (2020).

[38] Blossfeld H-P Rossbach H-G Von Maurice J. Education as a lifelong process: the German National Educational Panel Study (NEPS). Zeitschrift für Erziehungswissenschaft (ZfE), Sonderheft = Special issue 14, 2011 ed (2011).

[39] De Bock F Fischer JE Hoffmann K Renz-Polster H. A participatory parent-focused intervention promoting physical activity in preschools: design of a cluster-randomized trial. BMC Public Health. (2010) 10:49. 10.1186/1471-2458-10-49 20113522 PMC2835684

[40] Ganzeboom HBG De Graaf PM Treiman DJ. A standard international socio-economic index of occupational status. Soc Sci Res. (1992) 21:1–56. 10.1016/0049-089X(92)90017-B25320249

[41] Ganzeboom HB. A *new International Socio-Economic Index (ISEI) of Occupational Status for the International Standard Classification of Occupation* 2008. (ISCO-08) Constructed with Data from the ISSP 2002–2007. Annual Conference of International Social Survey Programme (2010). Lisbon.

[42] Maas CJM Hox JJ. Sufficient sample sizes for multilevel modeling. Methodology. (2005) 1:86–92. 10.1027/1614-2241.1.3.86

[43] Hox J McNeish D. “Small Samples in Multilevel Modeling,” In:Rens van de Schoot Miočević M, editors. Small Sample Size Solutions. London: Routledge (2020). p. 215–25.

[44] Rattay P Blume M Wachtler B Wollgast L Spallek J Hoffmann S. Socioeconomic position and self-rated health among female and male adolescents: the role of familial determinants in explaining health inequalities results of the German KiGGS study. PLoS One. (2022) 17:e0266463. 10.1371/journal.pone.0266463 35390046 PMC8989218

[45] Tanaka C Hikihara Y Ohkawara K Tanaka S. Locomotive and non-locomotive activity as determined by triaxial accelerometry and physical fitness in Japanese preschool children. Pediatr Exerc Sci. (2012) 24:420–34. 10.1123/pes.24.3.420 22971558

[46] Herr RM Diehl K Schneider S Osenbruegge N Memmer N Sachse S. Which meso-level characteristics of early childhood education and care centers are associated with health, health behavior, and wellbeing of young children? findings of a scoping review. Int J Environ Res Public Health. (2021) 18:9. 10.3390/ijerph18094973 34067043 PMC8125417

[47] Gubbels JS Van Kann DH de Vries NK Thijs C Kremers SP. The next step in health behavior research: the need for ecological moderation analyses—an application to diet and physical activity at childcare. Int J Behav Nutr Phys Act. (2014) 11:52. 10.1186/1479-5868-11-52 24742167 PMC4002539

[48] Bradley RH. “*From Home to Day Care: Chaos in the Family/Child-Care Mesosystem*,” In:Evans GW Wachs TD, editors. Chaos and its Influence on Children's Development: An Ecological Perspective, New York, NY: American Psychological Association (2010) p. 135–53.

[49] Boonpleng W Park CG Gallo AM Corte C McCreary L Bergren MD. Ecological influences of early childhood obesity: a multilevel analysis. West J Nurs Res. (2013) 35:742–59. 10.1177/0193945913480275 23493675

[50] Park SH Park CG Bahorski JS Cormier E. Factors influencing obesity among preschoolers: multilevel approach. Int Nurs Rev. (2019) 66:346–55. 10.1111/inr.12513 31016729

[51] WHO. World Obesity Day: Understanding the social consequences of obesity. (2017). Available online at: https://www.euro.who.int/en/health-topics/noncommunicable-diseases/mental-health/news/news/2017/10/world-obesity-day-understanding-the-social-consequences-of-obesity2017 (accessed October, 2021).

[52] Richter M Dragano N. Micro, macro, but what about meso? the institutional context of health inequalitie*s. Int J Public Health*. (2018) 63:163–4. 10.1007/s00038-017-1064-4 29250722

[53] Guddal MH Stensland S Småstuen MC Johnsen MB Zwart JA Storheim K. Physical activity and sport participation among adolescents: associations with mental health in different age groups. results from the Young-HUNT study: a cross-sectional survey. BMJ Open. (2019) 9:e028555. 10.1136/bmjopen-2018-028555 31488476 PMC6731817

[54] Vliet J Gustafsson P Duchen K Nelson N. Social inequality and age-specific gender differences in overweight and perception of overweight among Swedish children and adolescents: A cross-sectional study health behavior, health promotion and society. BMC public health. (2015) 15:628. 10.1186/s12889-015-1985-x 26156095 PMC4496810

[55] Shah B Tombeau Cost K Fuller A Birken CS Anderson LN. Sex and gender differences in childhood obesity: contributing to the research agenda. BMJ Nutri Prevent Health. (2020) 3:387–90. 10.1136/bmjnph-2020-000074 33521549 PMC7841817

[56] Telama R Yang X Leskinen E Kankaanpää A Hirvensalo M Tammelin T. Tracking of physical activity from early childhood through youth into adulthood. Med Sci Sports Exerc. (2014) 46:955–62. 10.1249/MSS.0000000000000181 24121247

[57] Bundeszentrale für gesundheitliche Aufklärung (BZgA). Sonderheft 03: Nationale Empfehlungen für Bewegung und Bewegungsförderung (2016).

[58] Friel S Chopra M Satcher D. Unequal weight: equity oriented policy responses to the global obesity epidemic. BMJ. (2007) 335:1241–3. 10.1136/bmj.39377.622882.47 18079548 PMC2137064

[59] Jansen PW Mensah FK Nicholson JM Wake M. Family and neighbourhood socioeconomic inequalities in childhood trajectories of BMI and overweight: longitudinal study of Australian children. PLoS One. (2013) 8:e69676. 10.1371/journal.pone.0069676 23936075 PMC3720589

[60] Herr RM De Bock F Diehl K Wiedemann E Sterdt E Blume M. Associations of individual factors and early childhood education and care (ECEC) centres characteristics with preschoolers' BMI in Germany. BMC Public Health. (2022) 22:1415. 10.1186/s12889-022-13814-5 35883054 PMC9317063

[61] Rosario AS Kurth BM Stolzenberg H Ellert U Neuhauser H. Body mass index percentiles for children and adolescents in Germany based on a nationally representative sample (KiGGS 2003-2006). Eur J Clin Nutr. (2010) 64:341–9. 10.1038/ejcn.2010.8 20179728

[62] Schneider S Diehl K Görig T Schilling L De Bock F Hoffmann K. Contextual influences on physical activity and eating habits -options for action on the community level. BMC Public Health. (2017) 17:760. 10.1186/s12889-017-4790-x 28964266 PMC5622514

[63] Jackson JK Grady A Lecathelinais C Fielding A Yoong SL. Parent-reported compared with researcher-measured child height and weight: impact on body mass index classification in Australian pre-school aged children. Health Promot J Austr. (2023) 10.1002/hpja.702 36734513 PMC10946955

[64] Huybrechts I Himes JH Ottevaere C De Vriendt T De Keyzer W Cox B. Validity of parent-reported weight and height of preschool children measured at home or estimated without home measurement: a validation study. BMC Pediatrics. (2011) 11:63. 10.1186/1471-2431-11-63 21736757 PMC3149571

[65] Schmutz EA Leeger-Aschmann CS Radtke T Muff S Kakebeeke TH Zysset AE. Correlates of preschool children's objectively measured physical activity and sedentary behavior: a cross-sectional analysis of the SPLASHY study. Int J Behav Nutri Physic Activ. (2017) 14:1. 10.1186/s12966-016-0456-9 28057008 PMC5216527

